# The Association Between Depression and Idiopathic Pulmonary Fibrosis: A Prospective Study in the UK Biobank

**DOI:** 10.1007/s44197-026-00541-y

**Published:** 2026-03-28

**Authors:** Qiao Yu, Jiaxi Pu, Fang Cao, Qiongjing Yuan, Junxia Yan, Pan Yu

**Affiliations:** 1 Department of Geriatric Medicine, Xiangya Hospital of Central South University, Changsha, 410008 China; 2 National Clinical Research Center for Geriatric Disorders, Xiangya Hospital of Central South University, Changsha, 410008 China; 3 Department of Nephrology, Xiangya Hospital of Central South University, Changsha, Hunan 410008 China; 4 Hunan Key Laboratory of Organ Fibrosis, Central South University, Changsha, Hunan 410008 China; 5 School of Public Health, Changsha Medical University, Changsha, Hunan 410219 China; 6 Department of Epidemiology and Health Statistics & Hunan Provincial Key Laboratory of Clinical Epidemiology, XiangYa School of Public Health, Central South University, Changsha, Hunan 410013 China

**Keywords:** Depression, Idiopathic pulmonary fibrosis, Potiential mediators

## Abstract

**Background:**

Although depression has been associated with various chronic diseases, its relationship with idiopathic pulmonary fibrosis (IPF) remains unclear.

**Methods:**

We included 353,855 participants from the UK Biobank. Depression was defined as (1) clinically diagnosed depression based on linked hospital records and self-reported physician diagnosis, and (2) depressive symptoms assessed by the Patient Health Questionnaire-2 (PHQ-2). We used Cox proportional hazards models to estimate hazard ratios (HRs) and 95% confidence intervals (CIs) for the association between depression and incident IPF. Causal mediation analysis was conducted using the R ‘mediation’ package to identify potential mediators of the depression-IPF association.

**Results:**

During a mean follow-up period of 14.5 years, we identified 2,261 new cases of IPF among the 353,855 participants in the study. Individuals with depression had a 52% higher risk of developing IPF compared to those without depression (HR: 1.52; 95% CI: 1.33–1.73). Mediation analysis indicated that aging, metabolism, obesity, and inflammation significantly mediated the relationship between depression and the risk of IPF (*p* < 0.05 for all).

**Conclusions:**

Our findings suggest that individuals with depression are at an elevated risk of IPF. The association between depression and IPF appears to be partially mediated by factors such as aging, metabolism, obesity, and inflammation.

**Supplementary Information:**

The online version contains supplementary material available at 10.1007/s44197-026-00541-y.

## Introduction

Depressive disorders represent a leading and growing cause of disability worldwide, affecting over 264 million individuals globally [1]. Growing evidence suggests that depression is associated with an increased risk of various adverse health outcomes, including rapid decline in kidney function [2], lung and smoking-related cancers [3], cardiovascular diseases [4], and both all-cause and cardiovascular disease-related mortality [5].

Idiopathic pulmonary fibrosis is recognized as the most common form of idiopathic interstitial pneumonia, characterized by a chronic and progressive fibrotic process in the lungs with an unknown etiology and associated with a poor prognosis [6, 7]. The scarcity of therapeutic options for IPF leads to a median life expectancy of just 2 to 4 years for patients diagnosed with the condition [6]. The incidence of IPF is indeed on the rise globally, with a consistent trend observed across various countries in recent years [8]. This increasing prevalence is accompanied by a concurrent rise in IPF-related hospitalization rates and mortality, which are significant contributors to the financial strain on healthcare systems [7, 9]. Although the definitive cause of IPF remains unclear, several non-genetic risk factors have been identified, such as advanced age, male gender, and a history of smoking. Evidence from observational studies indicate that conditions such as gastroesophageal reflux, obstructive sleep apnea, exposure to air pollution, herpesvirus infections, and specific occupational exposures are associated with an increased risk of interstitial lung diseases, including IPF [7]. Currently, there seems to be a scarcity of studies investigating the association between depression and the risk of IPF, leaving this relationship to be elucidated.

Previous studies have identified depression as being associated with various factors such as aging [10], metabolism [11], obesity [12], and inflammation [13]. These factors are also recognized for their close relationship with interstitial lung diseases, including IPF [14–17]. The connection between depression and these factors suggests a complex interplay that may contribute to the development and progression of IPF, highlighting the importance of considering mental health in the management of pulmonary diseases.

Previous research has primarily focused on depression as a comorbidity affecting prognosis in established IPF, leaving the association between depression and the subsequent development of IPF largely unexplored. In this study, our primary objective was to examine the correlation between depression and the risk of developing IPF and to investigate the potential mediators between depression and IPF within a cohort of 353,855 participants from the UK Biobank. Furthermore, we conducted an exploratory analysis to evaluate whether treatment with antidepressants and/or psychotherapy was associated with IPF risk in individuals with depression.

## Methods

### Study Design and Participants

**Fig. 1:**
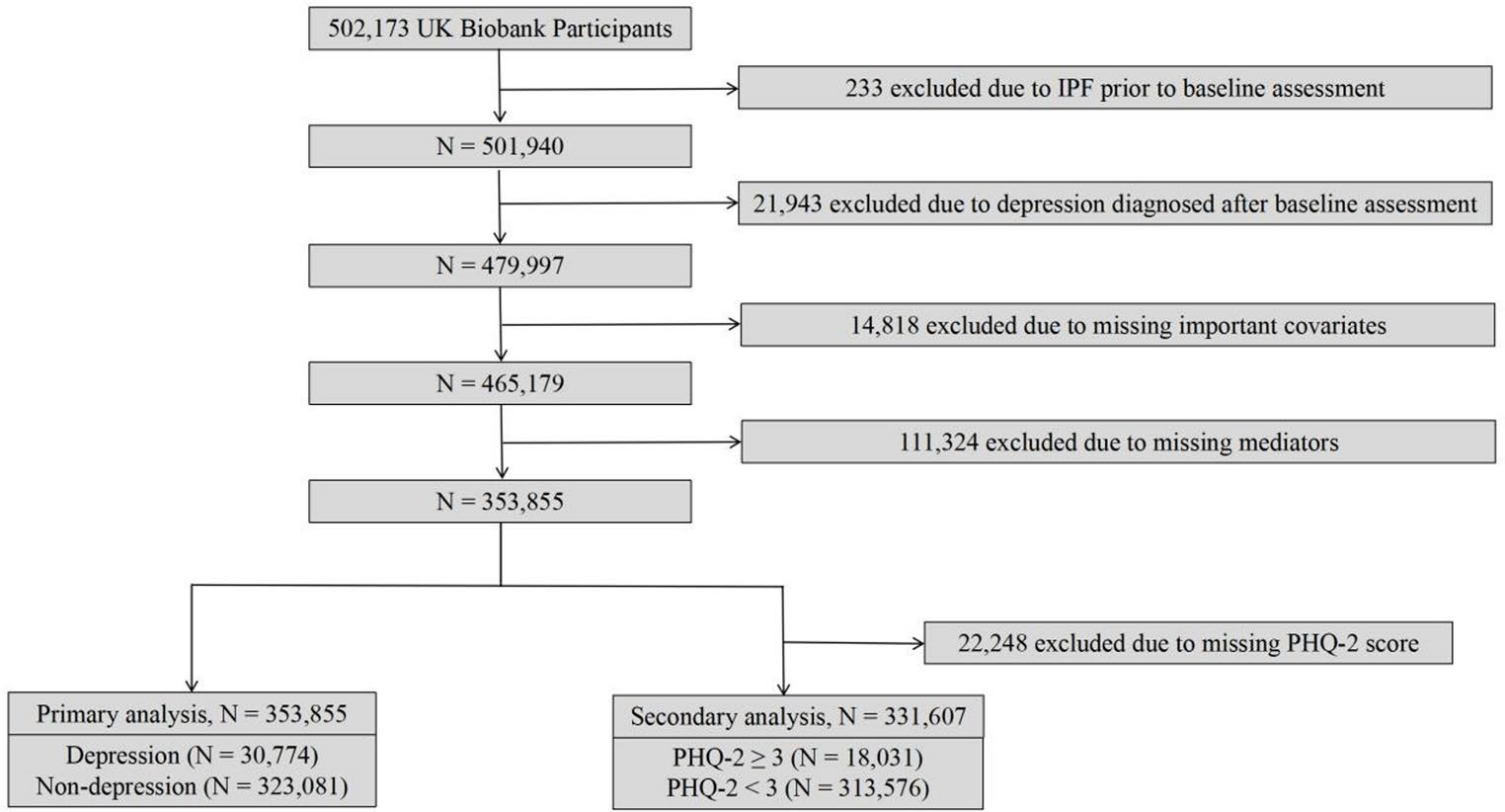
Flowchart of the participant selection process

Participants for this study were recruited from various regions across the United Kingdom between 2006 and 2010, constituting a total of 502,173 individuals from the UK Biobank, aged between 40 and 69 years. The participant selection process is detailed in Fig. 1. Briefly, from the initial cohort of 502,173 individuals, we sequentially excluded participants for the following reasons: (1) a history of IPF prior to baseline (*n* = 233); (2) a diagnosis of depression post-baseline (*n* = 21,943); (3) incomplete data for necessary covariates (*n* = 14,818); and (4) missing data on mediator variables (*n* = 111,324). This resulted in a final analytical sample of 353,855 participants for the primary analysis, including 30,774 with depression and 323,081 without. For the secondary analysis, an additional 22,248 participants without a baseline Patient Health Questionnaire-2 (PHQ-2) score were excluded, yielding a sample of 331,607 participants (18,031 with PHQ-2 scores ≥ 3 and 313,576 with PHQ-2 scores < 3). A detailed depiction of the participant selection process is presented in Fig. 1. The UK Biobank data were approved by the North West Multicenter Research Ethics Committee (MREC). Written informed consent was obtained from all the UK Biobank study participants.

### Exposure Assessment

This study employed a dual-method approach to identify cases of depression. First, we utilized linked hospital admission records to detect participants with a primary or secondary diagnosis of depression, classified according to ICD-10 codes F32 (major depressive disorder, single episode) and F33 (major depressive disorder, recurrent). In addition, the PHQ-2 was administered at baseline to assess for depressive symptoms. This brief 2-item tool assesses the frequency of a depressed mood and anhedonia experienced over the past two weeks, with response options ranging from “not at all” to “nearly every day” corresponding to scores of 0 to 3, respectively. Consequently, the PHQ-2 score ranges from 0 to 6, with a score of 3 or above indicating a potential depressive disorder.

### Outcomes Assessment

In this study, the outcome of interest was IPF, which was identified through the International Classification of Diseases, 10th Revision (ICD-10) code J84.1. The UK Biobank Outcome Adjudication Group, working in collaboration with clinical specialists, devised and validated algorithms that utilized clinical code lists to confirm a spectrum of health outcomes. Individuals were followed up from the date of recruitment to the date of IPF events, death, lost to follow up or the end of follow-up in April 2024, whichever occurred first.

### Mediators Assessment

The primary mediator variables in this study encompassed Phenotype age acceleration (PhenoAgeAccel), leucocyte telomere length (LTL), metabolic syndrome (MetS), body mass index (BMI), waist-to-hip ratio (WHR), and C-reactive protein (CRP). Phenotype age was established using mortality risk scores derived from the Gompertz proportional hazards model, which integrates chronological age with nine clinical chemistry biomarkers, and was crafted to predict all-cause mortality using data from the National Health and Nutrition Examination Survey (NHANES) III [18]. Subsequently, we calculated PhenoAgeAccel as the residuals from a linear regression of phenotypic age on chronological age. PhenoAgeAccel > 0 and ≤ 0 were defined as biologically older and younger than chronological age, respectively. LTL assessments were performed on DNA samples obtained at baseline, adhering to quality control procedures as previously described [19]. In brief, LTL was measured using a validated quantitative polymerase chain reaction (qPCR) method and was expressed as the ratio of telomere repeat sequences (T) to a single-copy gene (S), known as the T/S ratio. To normalize the data distribution, the T/S ratios were log-transformed using the natural logarithm (base e). To facilitate comparison with other datasets, we utilized z-score standardized LTL values, as detailed in the cited reference [19]. MetS is characterized by the presence of three or more of the following criteria: unhealthy waist circumference (WC), hypertension, dyslipidemia, hypertriglyceridemia, and hyperglycemia [20]. In this study, we used established reference values to define an unhealthy waist circumference, with the thresholds set at ≥ 88 cm for women and ≥ 102 cm for men. Hypertension was determined by a systolic blood pressure of ≥ 130 mmHg or a diastolic blood pressure of ≥ 85 mmHg. Dyslipidemia was identified by high-density lipoprotein (HDL) cholesterol levels < 1.036 mmol/L for men and < 1.295 mmol/L for women. Hypertriglyceridemia was characterized by triglyceride levels of ≥ 1.695 mmol/L. Hyperglycemia was diagnosed with fasting blood glucose (FBG) levels of ≥ 5.55 mmol/L [21]. For each participant, body weight and height were measured, which were then used to compute the BMI by dividing the body weight in kilograms by the square of the body height in meters (kg/m^2^). Waist and hip circumferences were also assessed with a measuring tape, and these measurements were utilized to determine the WHR.

### Covariate Assessment

To control for potential confounding factors in the relationship between depression and the risk of IPF, a variety of sociodemographic variables were included as covariates in our analysis. These covariates included age (continuous), sex (male or female), ethnicity (white or non-white), educational attainment (college/university degree or not), employment status (employed or unemployed), smoking history (never, former, or current), alcohol consumption (never, former, or current), and the Townsend Deprivation Index (TDI, continuous). The TDI is a comprehensive measure that captures data on social class, employment, and housing conditions, thereby reflecting the socioeconomic status (SES) of participants based on their geographical area. A higher TDI score indicates a greater degree of deprivation [22]. For categorical covariates, observations with missing data (e.g., responses of “Prefer not to answer” or “Do not know”) were excluded from the analyses to ensure the accuracy and reliability of the results.

### Statistical Analysis

In this study, we provided a detailed demographic profile by expressing continuous variables as mean values with their corresponding standard deviations (SD) and categorical variables as proportions (percentages). For group comparisons, continuous variables were analyzed using the unpaired Student’s t-test, while categorical variables were assessed with the chi-squared (χ²) test. We utilized Kaplan-Meier analysis and Cox proportional hazard models to investigate the association between depression, its treatment, and the risk of IPF. To account for potential confounding factors associated with IPF, we applied two multivariate models with incremental adjustments. Model 1 was adjusted for age and sex, while Model 2 incorporated the covariates from Model 1 and further included ethnicity, education, employment, smoking status, alcohol status, TDI, asthma, COPD, and the bronchiectasis. To clarify the link between depression and IPF, we explored both direct associations and indirect associations potentially mediated by PhenoAgeAccel, LTL, MetS, BMI, WHR, and CRP. By employing a parametric regression approach using the “mediation” package in R version 4.4.1, we could quantify the total effect of depression on IPF, as well as the natural direct effects (NDE) and natural indirect effects (NIE). The NDE captured the impact of depression on IPF not explained by the mediators, while the NIE represented the effect mediated through them. The proportion mediated was calculated as the ratio of the NIE to the total effect. To ensure robust statistical inference, non-parametric bootstrapping with 500 simulations was employed to calculate the 95% confidence intervals. All statistical analyses were conducted using R version 4.4.1, with statistical significance defined as a two-tailed *p*-value less than 0.05.

### Sensitivity Analysis

To ensure the robustness of our findings, we performed a series of sensitivity analyses as follows:

1. Exclusion based on follow-up duration: We excluded participants with a follow-up period of 5/10 years or less to mitigate the risk of reverse causation.
2. Additional adjustments: We further refined our model by incorporating additional adjustments for forced expiratory volume in 1 s (FEV1), particulate matter with an aerodynamic diameter less than 2.5 μm (PM2.5), and physical exercise, in addition to the covariates in Model 2.
3. Stratification analyses: We conducted stratification analyses to investigate the relationship between depression and the risk of IPF across strata of age, sex, ethnicity, education, employment, smoking status, alcohol status, TDI, and BMI.
4. Exclusion based on respiratory history: We explored the association between depression and IPF by limiting the analysis to participants without a history of chronic obstructive pulmonary disease (COPD), asthma, or bronchiectasis.

## Results

### Characteristics of participants

The baseline characteristics of the 353,855 participants, stratified by depression status, are detailed in Table 1. Throughout a mean follow-up period of 14.5 years, 2,261 (0.6%) participants developed incident IPF. Those with depression were more likely to be younger, female, and white; to have lower educational attainment, be unemployed, and have a higher TDI; and to smoke more and consume alcohol less frequently. In terms of mediating variables, participants with depression showed elevated levels of PhenoAgeAccel, LTL, BMI, CRP, and a higher rate of MetS. Furthermore, participants with depression had increased comorbidities of COPD, asthma, and bronchiectasis at baseline. Notably, the incidence of IPF during follow-up was significantly higher among participants with depression than in those without depression.

**Table 1:** Baseline characteristics of participants of UK Biobank Study by depression

Variables	Overall (*N* = 353,855)	Non-depression (*N* = 323,081)	Depression (*N* = 30,774)	*p* value	SMD
Age, years	56.55 ± 8.07	56.64 ± 8.09	55.56 ± 7.83	< 0.001	0.136
Sex, n (%)				< 0.001	0.259
Female	188,958 (53.4)	168,974 (52.3)	19,984 (64.9)		
Male	164,897 (46.6)	154,107 (47.7)	10,790 (35.1)		
Ethnicity, n (%)				< 0.001	0.085
Non-white	18,721 (5.3)	17,591 (5.4)	1130 (3.7)		
White	335,134 (94.7)	305,490 (94.6)	29,644 (96.3)		
Education, n (%)				< 0.001	0.052
Other	184,098 (52.0)	167,356 (51.8)	16,742 (54.4)		
College degree	169,757 (48.0)	155,725 (48.2)	14,032 (45.6)		
Employment, n (%)				< 0.001	0.175
Non-employed	147,829 (41.8)	132,540 (41.0)	15,289 (49.7)		
Employed	206,026 (58.2)	190,541 (59.0)	15,485 (50.3)		
Smoking status, n (%)				< 0.001	0.193
Never	194,781 (55.0)	179,850 (55.7)	14,931 (48.5)		
Previous	123,249 (34.8)	112,300 (34.8)	10,949 (35.6)		
Current	35,825 (10.1)	30,931 (9.6)	4894 (15.9)		
Alcohol status, n (%)				< 0.001	0.110
Never	14,873 (4.2)	13,444 (4.2)	1429 (4.6)		
Previous	12,089 (3.4)	9897 (3.1)	2192 (7.1)		
Current	326,893 (92.4)	299,740 (92.8)	27,153 (88.2)		
TDI, n (%)	−1.38 ± 3.03	−1.43 ± 3.00	−0.86 ± 3.26	< 0.001	0.185
PhenoAge age, years	50.48 ± 10.13	50.51 ± 10.14	50.17 ± 10.03	< 0.001	0.033
PhenoAgeAccel	0.00 ± 5.71	−0.07 ± 5.65	0.72 ± 6.29	< 0.001	0.132
LTL	0.00 ± 0.98	0.00 ± 0.98	0.01 ± 0.98	0.217	0.007
BMI, kg/m^2^	27.34 ± 4.69	27.26 ± 4.61	28.23 ± 5.42	< 0.001	0.193
WHR	0.872 ± 0.090	0.872 ± 0.090	0.871 ± 0.091	0.012	0.015
MetS, n (%)	96,916 (27.4)	86,808 (26.9)	10,108 (32.8)	< 0.001	0.131
Unhealthy WC, n (%)	116,991 (33.1)	103,922 (32.2)	13,069 (42.5)	< 0.001	0.214
Hypertension, n (%)	236,549 (66.8)	217,727 (67.4)	18,822 (61.2)	< 0.001	0.130
Dyslipidemia, n (%)	75,397 (21.3)	67,230 (20.8)	8167 (26.5)	< 0.001	0.135
Hypertriglyceridemia, n (%)	141,818 (40.1)	128,146 (39.7)	13,672 (44.4)	< 0.001	0.097
Hyperglycemia, n (%)	57,456 (16.2)	52,335 (16.2)	5121 (16.6)	0.045	0.012
CRP, mg/L	0.25 ± 0.43	0.25 ± 0.42	0.30 ± 0.47	< 0.001	0.122
Asthma, n (%)				< 0.001	0.142
No	314,649 (88.9)	288,628 (89.3)	26,021 (84.6)		
Yes	39,206 (11.1)	34,453 (10.7)	4753 (15.4)		
COPD, n (%)				< 0.001	0.110
No	348,169 (98.4)	318,349 (98.5)	29,820 (96.9)		
Yes	5686 (1.6)	4732 (1.5)	954 (3.1)		
Bronchiectasis, n (%)				< 0.001	0.024
No	352,755 (99.7)	322,118 (99.7)	30,637 (99.6)		
Yes	1100 (0.3)	963 (0.3)	137 (0.4)		
IPF, n (%)				< 0.001	0.031
No	351,594 (99.4)	321,091 (99.4)	30,503 (99.1)		
Yes	2261 (0.6)	1990 (0.6)	271 (0.9)		

Abbreviations: TDI, townsend deprivation index; PhenoAgeAccel, Phenotype age acceleration; LTL, leucocyte telomere length; BMI, body mass index; WHR, waist-to-hip ratio; MetS, metabolic syndrome; WC, waist circumference; CRP, C-reactive protein; COPD, chronic obstructive pulmonary disease; IPF, idiopathic pulmonary fibrosis.

### Associations Between Depression and Risks of Idiopathic Pulmonary fibrosis

**Fig. 2:**
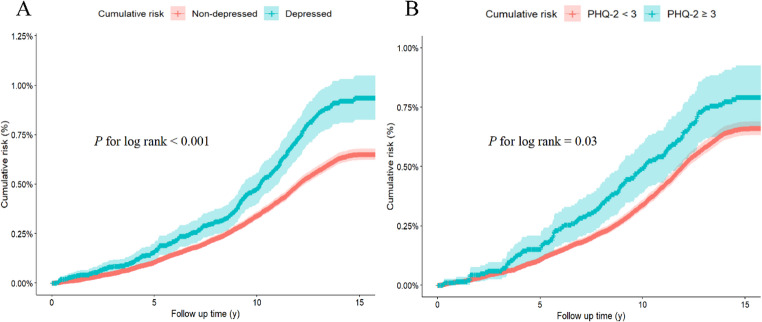
Cumulative incidence of idiopathic pulmonary fibrosis in relation to (**A**) Depressive Status and (**B**) Baseline PHQ-2 Score Among UK Biobank Participants

Figure 2A illustrates that participants with depression had a significantly higher cumulative incidence of IPF than those without depression (log-rank *p* < 0.001). Similarly, participants with a PHQ-2 score of 3 or higher showed a higher cumulative incidence of IPF compared to those with a score below 3 (log-rank *p* = 0.03) in Fig. 2B. Subsequently, we performed Cox proportional hazards regression analysis using three progressively adjusted models to evaluate the risk of developing IPF. After adjusting for sociodemographic factors in Model 2, participants with depression and a PHQ-2 score of 3 or higher were independently associated with an increased risk of developing IPF. The HRs were 1.58 (95% CI: 1.39 to 1.80) and 1.37 (95% CI: 1.14 to 1.63), respectively (Table 2).

**Table 2:** Associations of depression with idiopathic pulmonary fibrosis

Variables	Cumulative incidence	Unadjusted		Model 1		Model 2	
		HR (95% CI)	*p* value	HR (95% CI)	*p* value	HR (95% CI)	*p* value
Depression							
No	0.6%	Ref		Ref		Ref	
Yes	0.9%	1.44 (1.27 −1.64)	*p* < 0.001	1.81 (1.59–2.05)	*p* < 0.001	1.52 (1.33–1.73)	*p* < 0.001
PQH-2 ≥ 3							
No	0.7%	Ref		Ref		Ref	
Yes	0.8%	1.21 (1.02–1.44)	*p* = 0.034	1.67 (1.40–1.99)	*p* < 0.001	1.28 (1.07–1.53)	*p* = 0.007
Continuous PHQ-2		1.07 (1.03–1.11)	*p* < 0.001	1.20 (1.16–1.25)	*p* < 0.001	1.12 (1.08–1.16)	*p* < 0.001

Model 1: adjusted for age, sex

Model 2 (Primary model): adjusted for age, sex, ethnicity, education, employment, smoking status, alcohol status, TDI, Asthma, COPD, Bronchiectasis

### Potential Mediators Between Depression and Idiopathic Pulmonary fibrosis

**Fig. 3:**
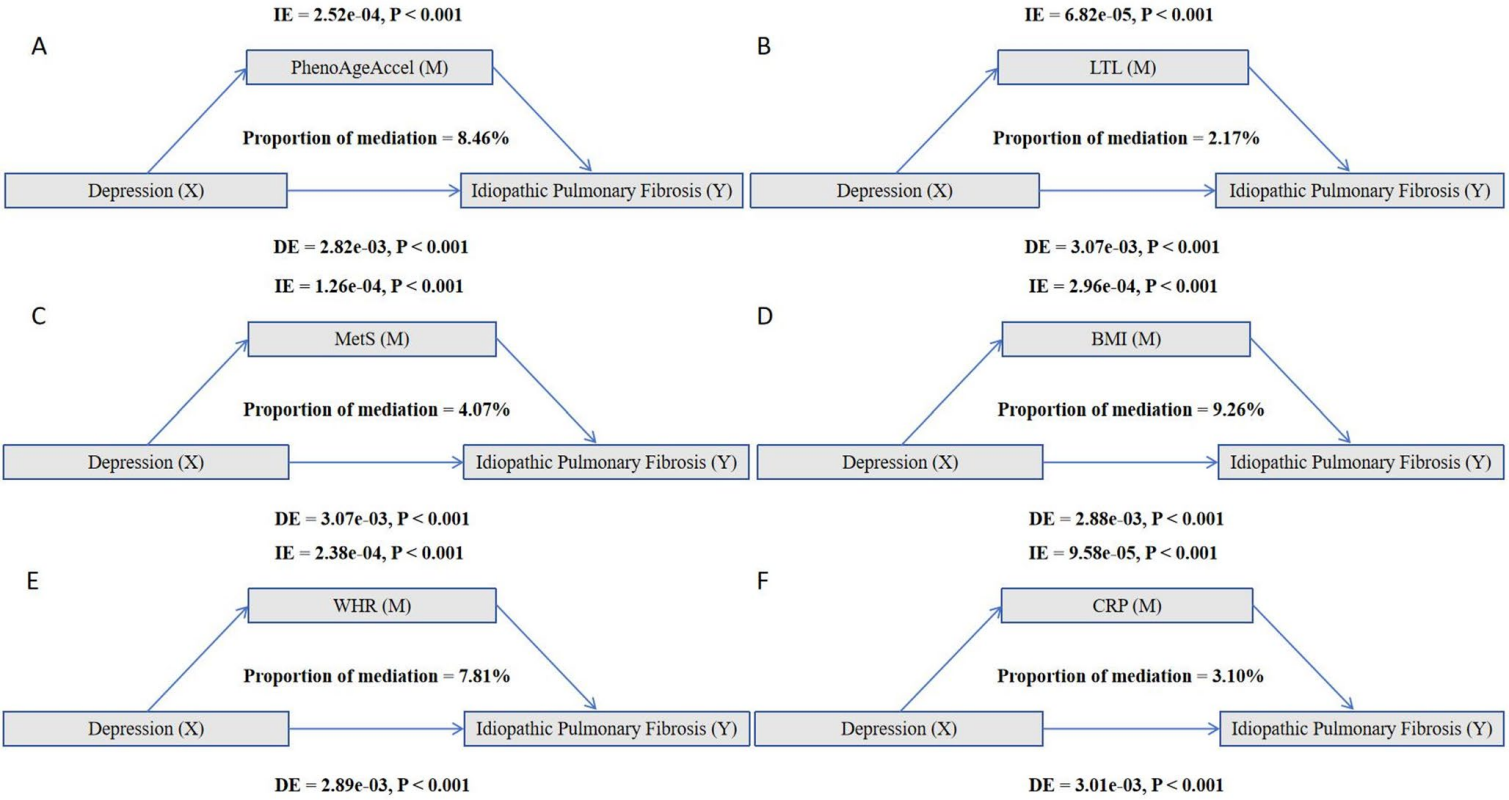
Potential mediators between depression and the occurrence of idiopathic pulmonary fibrosis. The model was adjusted for age, sex, ethnicity, education, employment, smoking status, alcohol status, TDI, Asthma, COPD, Bronchiectasis. Abbreviations: PhenoAgeAccel, Phenotype age acceleration; LTL, leucocyte telomere length; MetS, metabolic syndrome; BMI, body mass index; WHR, waist-to-hip ratio; CRP, C-reactive protein. The bars represent the proportion of the total association between depression and idiopathic pulmonary fibrosis that is mediated by each respective factor. The percentages displayed on each bar indicate this proportion

The mediation analysis was performed to identify the potential mediators and their respective impacts on the relationship between depression and the development of IPF. Figure 3 graphically represents the total, direct, and indirect effects of depression on IPF, with the results detailed as follows. In the mediation analysis, we found that several factors mediated the association between depression and the risk of IPF. The ranking of mediators by their effect size was as follows: BMI (9.26%) was the most prominent mediator, followed by PhenoAgeAccel (8.46%), WHR (7.81%), CRP (3.10%), MetS (4.07%), and LTL (2.17%).

### Sensitivity Analysis

**Fig. 4:**
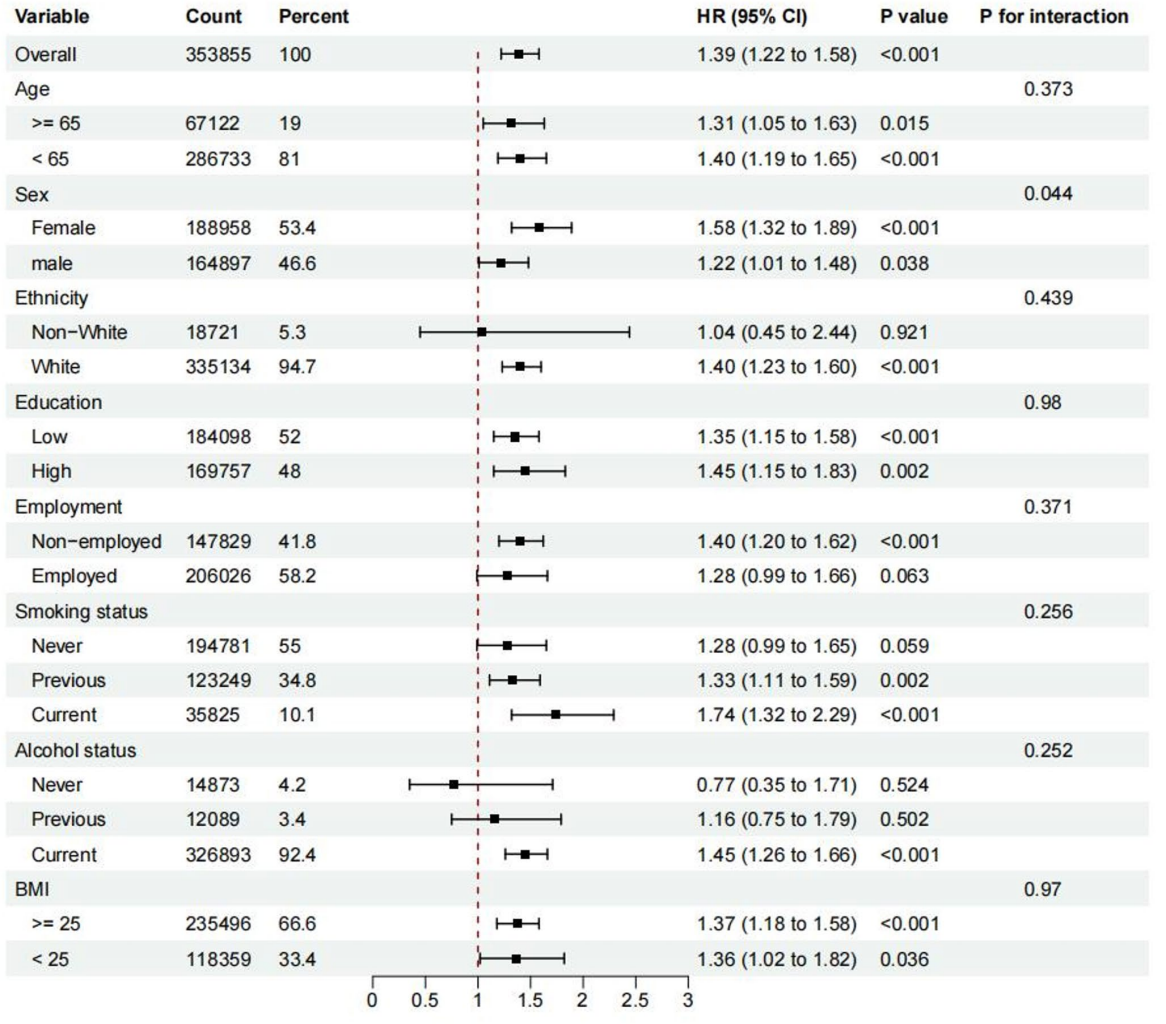
The association between depression with idiopathic pulmonary fibrosis by stratification analyses. The model was adjusted for age, sex, ethnicity, education, employment, smoking status, alcohol status, TDI, Asthma, COPD, Bronchiectasis

The robustness of the association between depression and IPF risk was confirmed through several sensitivity analyses: (1) Exclusion based on follow-up duration: To address potential reverse causation, we excluded participants with a follow-up of 5/10 years or less (Table S1). (2) Additional Adjustments: We further adjusted for additional variables—including FEV1, PM 2.5, and physical activity—on top of Model 2 (Table S2). (3) Stratification Analyses: We performed stratification analyses to further explore the association between depression and IPF risk (Fig. 4). (4) Exclusion based on respiratory history: We restricted the analysis to participants without a history of COPD, asthma, or bronchiectasis to assess the association (Table S3).

### An Exploratory Analysis Section

An exploratory analysis to investigate the associations between depression and IPF are shown in Table S4. After adjustment for demographic and risk factors in model 2, those receiving antidepressant treatment had a reduced risk of IPF (HR: 0.57, 95% CI: 0.40–0.82) compared to those not receiving antidepressant treatment (*p* = 0.001). Similarly, individuals who underwent psychotherapy also had a reduced risk of IPF (HR: 0.59, 95% CI: 0.39–0.88).

## Discussion

Our study reveals a significant association between depression and the risk of IPF. A pivotal finding is the identification of aging, metabolic syndrome, obesity, and inflammation as potential mediators in the relationship between depression and IPF onset. In the exploratory analysis, our study demonstrated that both antidepressant treatment and psychotherapy were significantly associated with lower risk of IPF in individuals with depression.

Although research on the link between depression and IPF remains limited, depression is prevalent among IPF patients, with reported rates ranging from 20% to 30% [23]. A Danish single-center study, which included 121 patients, underscored the prevalence of depression as a comorbid condition among individuals with IPF [24]. Despite the high prevalence of depressive symptoms in this population, these clinical manifestations have often been overlooked. Depression in IPF patients is consistently associated with a diminished quality of life (QoL). For instance, a prospective cohort study found that depressed IPF patients had a lower QoL than their non-depressed counterparts [25], and another study confirmed the significant impact of depression on health-related QoL in newly diagnosed patients [26]. Moreover, depression correlates with worse clinical outcomes, including higher St George’s Respiratory Questionnaire (SGRQ) scores, reduced 6-minute walk distances, and more persistent coughing [27]. The severity of depression has also been shown to be inversely correlated with key lung function measures, such as predicted FEV1 and forced vital capacity (FVC) [28]. These findings suggest that depression is intricately connected to the disease’s progression, the severity of symptoms, and the overall quality of life for patients with IPF, emphasizing the need for greater focus on the mental health of these individuals.

A recent study identified that among middle-aged and older Chinese individuals, depressive symptoms were associated with a higher risk of developing asthma and chronic lung disease [29]. However, to our knowledge, no research to date has definitively established a causal relationship between depression and IPF. Drawing on a large population with comprehensive follow-up, our study is the first to identify a positive association between depression and IPF risk, an association that persisted after stringent adjustment for confounders and rigorous sensitivity analyses. Although our study is observational by design, it provides preliminary evidence suggestive of a potential causal link. Further studies are crucial to validate the causal implications of our findings. Previous research has demonstrated the effectiveness of pulmonary rehabilitation programs, which include educational components, disease management strategies, and physical activities, in markedly improving exercise tolerance, reducing shortness of breath, and enhancing the QoL for individuals with IPF [25, 30, 31]. IPF patients may also experience significant benefits from cognitive behavioral therapy and professional counseling provided by healthcare professionals. Further research is warranted to determine whether psychological and pharmacological interventions for depression and anxiety can improve health outcomes in the IPF population, including QoL, mortality, and hospitalization rates [25]. Our findings suggest that antidepressant treatment and psychotherapy are associated with a lower risk of developing IPF. However, it is important to explicitly state that this finding cannot support a causal effect of treatment and is likely confounded by factors such as health-seeking behaviors or disease severity.

Our mediation analyses has shed light on the elevated risk of IPF associated with depression. Initially, we observed that various factors related to aging, such as PhenoAgeAccel and LTL, might partially account for the detrimental effects of depression on IPF [10, 14]. Research by Lorenzo and colleagues has synthesized evidence indicating that individuals with major depressive disorder (MDD) exhibit cellular and molecular changes linked to biological aging throughout their lives [10]. Aging is known to be associated with an increased vulnerability to interstitial lung diseases, with IPF being a prime example. Age-related changes, including genomic instability, telomere shortening, epigenetic modifications, cellular senescence, compromised autophagy, and mitochondrial dysfunction, may facilitate the development and progression of IPF [14]. Secondly, markers associated with MetS may partially mediate the relationship between depression and IPF. The link between depression and MetS is well-established by both observational [11, 32, 33] and Mendelian randomization [34] studies. Additionally, there is evidence linking MetS to various respiratory diseases, including asthma, COPD, obstructive sleep apnea, and pulmonary hypertension [15]. Furthermore, the triglyceride-glucose index (TyG), which serves as an indicator of metabolic dysfunction, has been associated with pulmonary health [35]. Thirdly, obesity appears to be a significant factor in the relationship between depression and IPF. Depression has been linked to an increased risk of obesity [12, 36], which in turn contributes to IPF development [16, 37]. Finally, the increased risk of IPF associated with depression may also be partially attributed to inflammation, aligning with previous research. There is a well-documented interplay between depression and inflammation, in a relationship where each condition can exacerbate the other [13, 38]. Studies have highlighted the role of inflammatory cells, especially neutrophils and macrophages, in the activation of profibrotic mediators that drive fibrogenesis [39]. Notably, a systematic review and meta-analysis demonstrated that treatment with selective serotonin inhibitors (e.g., fluoxetine) can significantly decrease levels of pro-inflammatory cytokines such as TNF-α, directly linking antidepressant use to the modulation of key mediators involved in fibrosis [40, 41].

A primary strength of our study lies in its comprehensive exploration of the prospective relationship between depression and IPF risk, and the potential mediators underlying these associations. This approach helps to fill significant gaps in our understanding of the complex interplay between depression and IPF risk. Additional study strengths include the large sample size, enhancing generalizability; the lengthy follow-up period, allowing for accurate assessment of long-term risks; and extensive adjustment for confounders, ensuring the robustness of the observed associations. The study also benefits from rigorous methods for evaluating depression and a thorough series of sensitivity analyses, confirming the reliability and stability of our findings.

This study has several limitations. First, its observational nature precludes causal inference, as the findings demonstrate an association, not causation, and we cannot exclude the possibility of reverse causation. Second, the differential detection rate could artificially inflate IPF incidence in the depression group, creating a spurious association. Diagnostic surveillance bias is a critical confounding factor for our findings. Third, our mediation analysis was limited to a single baseline mediator, due to the observational and cross-sectional nature of the data regarding the mediators, this study cannot establish temporal relationships, thereby precluding causal inferences within the mediation pathway. Fourth, we acknowledge the potential for “confounding by indication” in the treatment analysis, noting that patients receiving treatment may differ in severity, healthcare access, or other unmeasured factors from those who do not, which could influence our findings. Additionally, we have added a note regarding the lack of data on depression chronicity, severity, or specific antidepressant classes, which are important limitations of our dataset.

## Conclusion

Our study has demonstrated a significant association between depression and the risk of developing IPF. Notably, our findings suggest that aging, metabolic syndrome, obesity, and inflammation act as potential mediators in this relationship. In the exploratory analysis, patients with depression receiving antidepressants and psychotherapy showed a significantly lower risk of IPF. Randomized trials are needed to determine if effective depression treatment reduces incident IPF or attenuates progression in early disease.

## Supplementary Information

Below is the link to the electronic supplementary material.

Supplementary Material 1 (DOCX 18.7 KB)

Supplementary Material 2 (DOCX 14.3 KB)

Supplementary Material 3 (DOCX 14.1 KB)

Supplementary Material 4 (DOCX 12.9 KB)

## Data Availability

Data can be accessed at the UK Biobank (http://www.ukbiobank.ac.uk).
