# Supplementary material for: The Association Between Depression and Idiopathic Pulmonary Fibrosis: A Prospective Study in the UK Biobank
Source: J Epidemiol Glob Health. 2026 Mar 28;16(1):54. doi: 10.1007/s44197-026-00541-y (PMC13149822; doi:10.1007/s44197-026-00541-y)
Supplement: Supplementary file 1 — Supplementary Material 1 (DOCX 18.7 KB) [file 44197_2026_541_MOESM1_ESM.docx]

**Supplementary Table 1.1 Associations of depression with idiopathic pulmonary fibrosis by excluding participants with ≤ 5 years of follow-up**

|  | Unadjusted | | Model 1 | | Model 2 | |
| --- | --- | --- | --- | --- | --- | --- |
| Depression | HR (95% CI) | *p* value | HR (95% CI) | *p* value | HR (95% CI) | *p* value |
| No | Ref |  | Ref |  | Ref |  |
| Yes | 1.43 (1.25 - 1.65) | *p* < 0.001 | 1.80 (1.57 - 2.07) | *p* < 0.001 | 1.53 (1.33 - 1.77) | *p* < 0.001 |
| PQH-2 ≥ 3 |  |  |  |  |  |  |
| No | Ref |  | Ref |  | Ref |  |
| Yes | 1.17 (0.96 - 1.42) | *p* = 0.121 | 1.62 (1.33 - 1.97) | *p* < 0.001 | 1.27 (1.04 - 1.55) | *p* = 0.019 |
| Continuous PHQ-2 | 1.06 (1.02 - 1.10) | *p* = 0.007 | 1.19 (1.14 - 1.24) | *p* < 0.001 | 1.11 (1.07 - 1.16) | *p* < 0.001 |

Model 1: adjusted for age, sex

Model 2 (Primary model): adjusted for age, sex, ethnicity, education, employment, smoking status, alcohol status, TDI, Asthma, COPD, Bronchiectasis.

**Supplementary Table 1.2 Associations of depression with idiopathic pulmonary fibrosis by excluding participants with ≤ 10 years of follow-up**

|  | Unadjusted | | Model 1 | | Model 2 | |
| --- | --- | --- | --- | --- | --- | --- |
| Depression | HR (95% CI) | *p* value | HR (95% CI) | *p* value | HR (95% CI) | *p* value |
| No | Ref |  | Ref |  | Ref |  |
| Yes | 1.48 (1.23 - 1.78) | *p* < 0.001 | 1.86 (1.54 - 2.24) | *p* < 0.001 | 1.59 (1.32 - 1.92) | *p* < 0.001 |
| PQH-2 ≥ 3 |  |  |  |  |  |  |
| No | Ref |  | Ref |  | Ref |  |
| Yes | 0.92 (0.69 - 1.23) | *p* = 0.590 | 1.28 (0.96 - 1.71) | *p* = 0.098 | 1.05 (0.78 - 1.41) | *p* = 0.758 |
| 1-point change in PHQ-2 | 1.02 (0.97 - 1.08) | *p* = 0.405 | 1.15 (1.09 - 1.22) | *p* < 0.001 | 1.09 (1.03 - 1.15) | *p* < 0.001 |

Model 1: adjusted for age, sex

Model 2: adjusted for age, sex, ethnicity, education, employment, smoking status, alcohol status, TDI, Asthma, COPD, Bronchiectasis.
