# Supplementary material for: The Association Between Depression and Idiopathic Pulmonary Fibrosis: A Prospective Study in the UK Biobank
Source: J Epidemiol Glob Health. 2026 Mar 28;16(1):54. doi: 10.1007/s44197-026-00541-y (PMC13149822; doi:10.1007/s44197-026-00541-y)
Supplement: Supplementary file 2 — Supplementary Material 2 (DOCX 14.3 KB) [file 44197_2026_541_MOESM2_ESM.docx]

**Supplementary Table 2. Associations of depression with idiopathic pulmonary fibrosis by additionally adjusted with FEV1、PM 2.5 and physical exercise based on model 2**

|  | Unadjusted | | Model 1 | | Model 2 | |
| --- | --- | --- | --- | --- | --- | --- |
| Depression | HR (95% CI) | *p* value | HR (95% CI) | *p* value | HR (95% CI) | *p* value |
| No | Ref |  | Ref |  | Ref |  |
| Yes | 1.42 (1.15 - 1.76) | *p* = 0.001 | 1.83 (1.48 - 2.27) | *p* < 0.001 | 1.60 (1.29 - 1.99) | *p* < 0.001 |
| PQH-2 ≥ 3 |  |  |  |  |  |  |
| No | Ref |  | Ref |  | Ref |  |
| Yes | 0.95 (0.66 - 1.36) | *p* = 0.763 | 1.32 (0.92 - 1.89) | *p* = 0.132 | 1.04 (0.72 - 1.50) | *p* = 0.827 |
| Continuous PHQ-2 | 1.03 (0.96 - 1.10) | *p* = 0.457 | 1.17 (1.09 - 1.24) | *p* < 0.001 | 1.09 (1.02 - 1.16) | *p* = 0.016 |

Model 1: adjusted for age, sex

Model 2 (Primary model) : adjusted for age, sex, ethnicity, education, employment, smoking status, alcohol status, TDI, Asthma, COPD, Bronchiectasis, FEV1, PM 2.5, physical exercise.
