# Supplementary material for: The Association Between Depression and Idiopathic Pulmonary Fibrosis: A Prospective Study in the UK Biobank
Source: J Epidemiol Glob Health. 2026 Mar 28;16(1):54. doi: 10.1007/s44197-026-00541-y (PMC13149822; doi:10.1007/s44197-026-00541-y)
Supplement: Supplementary file 3 — Supplementary Material 3 (DOCX 14.1 KB) [file 44197_2026_541_MOESM3_ESM.docx]

**Supplementary Table 3. Associations of depression with idiopathic pulmonary fibrosis by restricting analysis to participants without self-reported histories of asthma, COPD, and bronchiectasis（N = 310758）**

|  | Unadjusted | | Model 1 | | Model 2 | |
| --- | --- | --- | --- | --- | --- | --- |
| Depression | HR (95% CI) | *p* value | HR (95% CI) | *p* value | HR (95% CI) | *p* value |
| No | Ref |  | Ref |  | Ref |  |
| Yes | 1.35 (1.16 - 1.57) | *p* < 0.001 | 1.69 (1.46 - 1.96) | *p* < 0.001 | 1.52 (1.30 - 1.77) | *p* < 0.001 |
| PQH-2 ≥ 3 |  |  |  |  |  |  |
| No | Ref |  | Ref |  | Ref |  |
| Yes | 1.10 (0.90 - 1.36) | *p* = 0.37 | 1.53 (1.24 - 1.89) | *p* < 0.001 | 1.29 (1.04 - 1.59) | *p* = 0.018 |
| Continuous PHQ-2 | 1.04 (0.99 - 1.08) | *p* = 0.112 | 1.17 (1.12 - 1.22) | *p* < 0.001 | 1.11 (1.07 - 1.16) | *p* < 0.001 |

Model 1: adjusted for age, sex

Model 2 (Primary model): adjusted for age, sex, ethnicity, education, employment, smoking status, alcohol status, TDI, .
