# Supplementary material for: The Association Between Depression and Idiopathic Pulmonary Fibrosis: A Prospective Study in the UK Biobank
Source: J Epidemiol Glob Health. 2026 Mar 28;16(1):54. doi: 10.1007/s44197-026-00541-y (PMC13149822; doi:10.1007/s44197-026-00541-y)
Supplement: Supplementary file 4 — Supplementary Material 4 (DOCX 12.9 KB) [file 44197_2026_541_MOESM4_ESM.docx]

Table S4. Associations of treatments with idiopathic pulmonary fibrosis in individuals with depression

| Treatment | Unadjusted | | Model 1 | | Model 2 | |
| --- | --- | --- | --- | --- | --- | --- |
|  | HR (95% CI) | *p* value | HR (95% CI) | *p* value | HR (95% CI) | *p* value |
| Antidepressants |  |  |  |  |  |  |
| No | Ref |  | Ref |  | Ref |  |
| Yes | 0.40 (0.28 - 0.57) | *p* < 0.001 | 0.46 (0.32 - 0.65) | *p* < 0.001 | 0.57 (0.40 - 0.82) | *p* = 0.002 |
| Psychotherapy |  |  |  |  |  |  |
| No | Ref |  | Ref |  | Ref |  |
| Yes | 0.39 (0.26 - 0.58) | *p* < 0.001 | 0.47 (0.32 - 0.70) | *p* < 0.001 | 0.59 (0.39 - 0.88) | *p* = 0.009 |

Model 1: adjusted for age, sex

Model 2 (Primary model): adjusted for age, sex, ethnicity, education, employment, smoking status, alcohol status, TDI, Asthma, COPD, Bronchiectasis.
